# Palivizumab coverage rates among moderate-to-late preterm infants in Korea: a nationwide cross-sectional study

**DOI:** 10.4178/epih.e2025015

**Published:** 2025-04-01

**Authors:** Seungyeon Kim, Saram Lee, Young June Choe, Ju Sun Heo

**Affiliations:** 1 College of Pharmacy, Dankook University, cheonan, Korea; 2 Department of Transdisciplinary Medicine, Seoul National University Hospital, Seoul, Korea; 3 Department of Medicine, Seoul National University College of Medicine, Seoul, Korea; 4 Innovative Medical Technology Research Institute, Seoul National University Hospital, Seoul, Korea; 5 Department of Pediatrics, Anam Hospital, Korea University College of Medicine, Seoul, Korea; 6 Department of Pediatrics, Seoul National University College of Medicine, Seoul, Korea; 7 Department of Pediatrics, Seoul National University Children’s Hospital, Seoul, Korea

**Keywords:** Premature infant, Respiratory syncytial virus, Prophylaxis, Vaccination coverage, Respiratory tract infections, Palivizumab

## Abstract

**OBJECTIVES:**

Since October 2016, Korea has implemented a national reimbursement program for palivizumab aimed at moderate-to-late preterm (MLPT) infants born between 32 0/7 weels and 35 6/7 weeks of gestation during the respiratory syncytial virus (RSV) season (October–March). However, large-scale data on coverage rates and associated factors remain limited. This study evaluated palivizumab coverage rates and identified predictive factors influencing its administration in MLPT infants.

**METHODS:**

This nationwide, population-based cross-sectional study utilized data from the Korean National Health Insurance Service collected between October 2016 and March 2019. MLPT infants eligible for palivizumab reimbursement were divided into administration and non-administration groups. Seasonal and overall coverage rates were assessed. A multivariate logistic regression analysis examined factors associated with palivizumab administration, with a focus on infant and maternal characteristics.

**RESULTS:**

Among 2,843 eligible MLPT infants, 1,201 (42.2%) received palivizumab, while 1,642 (57.8%) did not. Although coverage rates increased annually, they remained suboptimal. Lower palivizumab prophylaxis coverage was observed in infants with higher gestational ages, female sex, absence of low birth weight, those born in March, residents of non-capital areas, infants not admitted to a neonatal intensive care unit at birth, and infants of mothers aged <35 years.

**CONCLUSIONS:**

In the initial 3 RSV seasons following the introduction of palivizumab reimbursement for MLPT infants in Korea, the overall coverage rate was low (42.2%). National policies targeting infants with higher gestational ages, those born in March, and those residing in non-capital areas are necessary to improve coverage and ensure equitable RSV prophylaxis.

## GRAPHICAL ABSTRACT

[Fig f4-epih-47-e2025015]

## Key Message

• In Korea, following the introduction of palivizumab for MLPT infants, the overall coverage rate during the initial 3 RSV seasons was low (42.2%).

• Infants with higher gestational ages, those born in March, and residents of non-capital areas exhibited significantly lower coverage rates.

## INTRODUCTION

Respiratory syncytial virus (RSV) is the leading cause of acute lower respiratory infections (ALRIs) in infants and young children and significantly contributes to their morbidity and mortality [1-3]. Globally, in 2019, RSV accounted for 2.0% of deaths in children aged 0-60 months and 3.6% of deaths in those aged 28 days to 6 months [1]. In addition, early-life RSV-related ALRIs may be associated with long-term wheezing and asthma in childhood [4-6]. Preterm birth is a well-established risk factor for severe RSV-related ALRIs. Although the risk increases with decreasing gestational age (GA), moderate-to-late preterm (MLPT) infants born between 32 weeks and 35 weeks GA represent a substantial population at increased risk for severe RSV-related ALRIs [7-10]. A pooled analysis of 7 studies found that RSV hospitalization occurred in 3.7% of MLPT infants without comorbidities. Among these hospitalized infants, 82.0% required respiratory support, 10.2% required mechanical ventilation, and 17.9% required intensive care unit (ICU) admission [7]. MLPT infants born at 32-35 weeks GA comprise approximately 40% of all preterm infants [11]. Therefore, strategies to reduce the burden of RSV-related ALRIs in MLPT infants are needed.

As of the study period (October 2016 to March 2019), immunoprophylaxis with the RSV-neutralizing monoclonal antibody palivizumab was the most widely available strategy for preventing RSV-related ALRIs in high-risk infants. Recently, however, nirsevimab—a novel monoclonal antibody for RSV prevention—has been introduced and has gained widespread use in the United States and Europe since the final year of our study period. In Korea, national reimbursement for palivizumab in MLPT infants began in October 2016. For MLPT infants to be eligible for insurance coverage, they must be born during the RSV season (October to March) and have older siblings. Palivizumab is administered monthly, with a maximum of 5 doses allowed during the initial RSV season.

Assessing coverage rates provides valuable insights into the effectiveness of immunization programs and helps evaluate the potential spread of infectious diseases. This information is essential for developing strategies to enhance palivizumab prophylaxis coverage and serves as a basis for policy recommendations. Although the National Immunization Program in Korea monitors mandatory vaccination rates nationwide, palivizumab is not included in this program. Consequently, large-scale data on coverage rates and factors influencing palivizumab prophylaxis in MLPT infants remain scarce.

This study aimed to determine the coverage rate of palivizumab in MLPT infants following the implementation of palivizumab prophylaxis and to analyze predictive factors associated with non-administration using data from the Korean National Health Insurance Service (NHIS) database.

## MATERIALS AND METHODS

### Data source and study population

This nationwide, population-based cross-sectional study utilized the mother-offspring cohort derived from the Korean NHIS database [12]. The cohort, which includes all newborns and their mothers, was constructed and validated by the NHIS. An algorithm linked parents to their offspring using unique health insurance card numbers and delivery dates provided specifically for research purposes [13-16]. The NHIS mother-offspring cohort comprises comprehensive medical claims data, including socio-demographic information, healthcare service utilization (diagnoses, medical procedures, and prescriptions), and medical checkups for both adults and neonates, as well as death records. For adults, medical checkups also include lifestyle data such as smoking and alcohol consumption, whereas for neonates, details such as birth weight and feeding information are recorded. Our study analyzed data for all live births between October 1, 2016, and March 31, 2019, along with information on their mothers. Maternal delivery records from 2002 to 2020 were obtained from the NHIS database to identify newborn siblings.

Since the expansion of insurance coverage for palivizumab prophylaxis in Korea in 2016, the study population comprised all MLPT infants (32 0/7-35 6/7 weeks GA) eligible for palivizumab prophylaxis. Participants were excluded if they had conditions such as bronchopulmonary dysplasia, congenital heart disease, immunodeficiency, chronic respiratory abnormalities, neuromuscular disease, organ transplantation, or chromosomal abnormalities (e.g., Down syndrome). Diagnoses for these conditions were confirmed using International Classification of Diseases, 10th revision (ICD-10) diagnostic codes, with a list provided in Supplementary Material 1. Additionally, infants without older siblings or those not born during the RSV season (October to March) were excluded, as the insurance expansion specifically targeted MLPT infants born during the RSV season with older siblings. The presence of older siblings was verified by reviewing maternal delivery histories and comparing delivery dates with the infants’ birth dates. In cases of multiple births, newborns were classified as having no older siblings.

### Data collection

Drug prescription records were reviewed to determine whether infants received palivizumab for RSV prophylaxis during the first RSV season. Infants prescribed palivizumab at least once were classified as part of the palivizumab administration group.

Comprehensive data on the demographic characteristics of the infants and their mothers, as well as confounding factors associated with ALRI in preterm infants, were extracted to identify factors influencing palivizumab prophylaxis. Infant-related factors included GA, sex, and birth month (January, February, March, October, November, and December). Data on residential areas and birth years during the RSV seasons (October 2016 to March 2017, October 2017 to March 2018, or October 2018 to March 2019) were also collected. Baseline comorbidities recorded from birth to hospital discharge were classified as low GA, high GA, low birth weight, respiratory distress syndrome, sepsis, necrotizing enterocolitis, intraventricular hemorrhage, or retinopathy of prematurity (ROP). Neonatal intensive care unit (NICU) admission at birth was verified as well. The ICD-10 codes for these baseline comorbidities are provided in Supplementary Material 2.

Additionally, data on various maternal factors, including age, body mass index, socioeconomic status, type of insurance (medical insurance or Medical Aid), smoking status, and alcohol consumption, were collected.

### Statistical analysis

Descriptive statistics, Pearson’s chi-square test, and Student’s t-test were used to compare the baseline characteristics of neonates who received palivizumab prophylaxis with those who did not. Baseline characteristics were also analyzed separately for infants born at 35 weeks GA and those born at less than 35 weeks GA. Categorical variables are presented as frequencies with percentages, and continuous variables are presented as mean±standard deviation. Yearly trends in palivizumab administration during the RSV season for neonates with older siblings were analyzed by calculating and comparing coverage rates across the entire population and within subpopulations categorized by GA, specifically comparing infants born at ≥35 weeks GA to those born at <35 weeks GA. Additionally, the coverage rate of palivizumab prophylaxis was analyzed based on the residential areas of newborns in Korea. To improve clarity and readability, a column displaying palivizumab coverage rates as row percentages for each subgroup was added to Table 1.

Factors associated with palivizumab prophylaxis were identified using univariate and multivariate logistic regression analyses. Neonates prescribed palivizumab were compared with those who were not. The analyses included infant factors such as GA, sex, birth month and year, residential area, comorbidities, and NICU admission, as well as maternal factors such as insurance type and age. These factors were selected based on a review of relevant literature and clinical considerations regarding their potential influence on palivizumab prophylaxis. Residential areas were categorized as Seoul, greater Seoul (Incheon and Gyeonggi), and non-capital areas. Results are presented as odds ratios with corresponding 95% confidence intervals (CIs). Multivariable logistic models were adjusted for baseline characteristics that significantly differed between the palivizumab administration and non-administration groups. Logistic regression analyses were conducted separately for neonates born at 35 weeks GA and those born at 32-34 weeks GA.

All statistical analyses were performed using SAS version 9.4 (SAS Institute Inc., Cary, NC, USA), with statistical significance set at p-value<0.05.

### Ethics statement

The Institutional Review Board of Seoul National University Hospital approved this study (IRB No. E-2201-009-1286) and waived the requirement for informed consent due to the use of anonymized clinical data.

## RESULTS

### Study population

**Figure 1.**
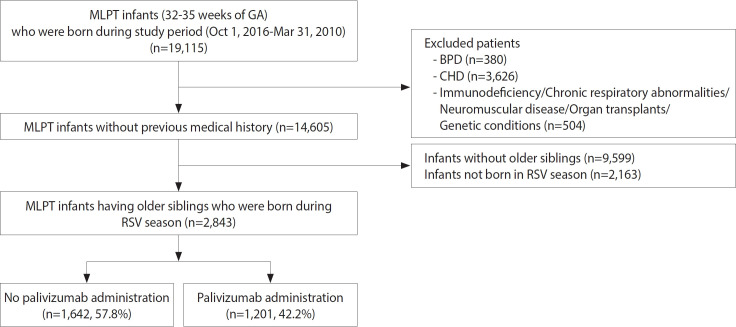
Flowchart of study population selection and palivizumab administration in moderate-to-late preterm (MLPT) infants. GA, gestational age; BPD, bronchopulmonary dysplasia; CHD, congenital heart disease; RSV, respiratory syncytial virus.

Among the 19,115 MLPT infants born between 32 0/7 weeks and 35 6/7 weeks GA, 4,510 were excluded due to underlying medical conditions, such as bronchopulmonary dysplasia, congenital heart disease, immunodeficiency, chronic respiratory abnormalities, neuromuscular disease, organ transplantation, and genetic disorders (Figure 1). Additionally, 9,599 infants without older siblings and 2,163 infants not born during the RSV season were excluded. Of the remaining 2,843 infants, 1,642 were assigned to the non-palivizumab group and 1,201 to the palivizumab group.

### Coverage rate of palivizumab

The overall palivizumab coverage rate was 42.2%. Coverage rates increased over time, from 37.8% during the October 2016 to March 2017 season, to 41.6% in the October 2017 to March 2018 season, and to 47.9% in the October 2018 to March 2019 season (Supplementary Material 3). The coverage rate in the October 2018 to March 2019 season was significantly higher than that in the October 2016 to March 2017 season, with a mean increase of 10.1%.

**Figure 2.**
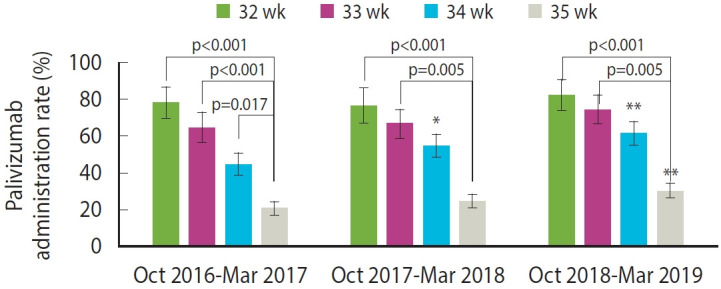
Yearly trend of palivizumab coverage rate by gestational age. *p<0.05, **p<0.01 compared to 2016 season.

Coverage rates by GA were as follows: 78.9% for 32 weeks GA, 68.4% for 33 weeks GA, 53.0% for 34 weeks GA, and 25.1% for 35 weeks GA. The coverage rate for the 32-34 weeks GA group was 62.0%, which was significantly higher than the rate for the 35 weeks GA group. This GA-related pattern of administration was observed consistently across all seasons (Figure 2). During the October 2016 to March 2017 season, infants born at 35 weeks GA received palivizumab at significantly lower rates than those born at 32 weeks, 33 weeks, or 34 weeks GA (p<0.001, <0.001, and 0.017, respectively). Moreover, in the October 2017 to March 2018 and October 2018 to March 2019 seasons, infants born at 35 weeks GA consistently exhibited significantly lower coverage rates than those born at 32 weeks and 33 weeks GA (p<0.001, 0.005, respectively). These trends were consistently observed when comparing yearly palivizumab coverage rates between 35 weeks GA infants and those in the 32-34 weeks GA group (Supplementary Material 4). The administration rate was significantly different between 35 weeks GA infants and 32-34 weeks GA infants across seasons (October 2016 to March 2017: 56.0 vs. 20.8%; October 2017 to March 2018: 62.1 vs. 24.7%; October 2018 to March 2019: 69.0 vs. 30.3%; all p<0.001). Additionally, within the same GA groups, administration rates differed significantly between the October 2016 to March 2017 season and the October 2018 to March 2019 season (for 35 weeks GA infants, 20.8 vs. 30.3%; for 32-34 weeks GA infants, 56.0 vs. 69.0%; all p<0.001).

### Factors associated with low coverage rate of palivizumab

**Figure 3.**
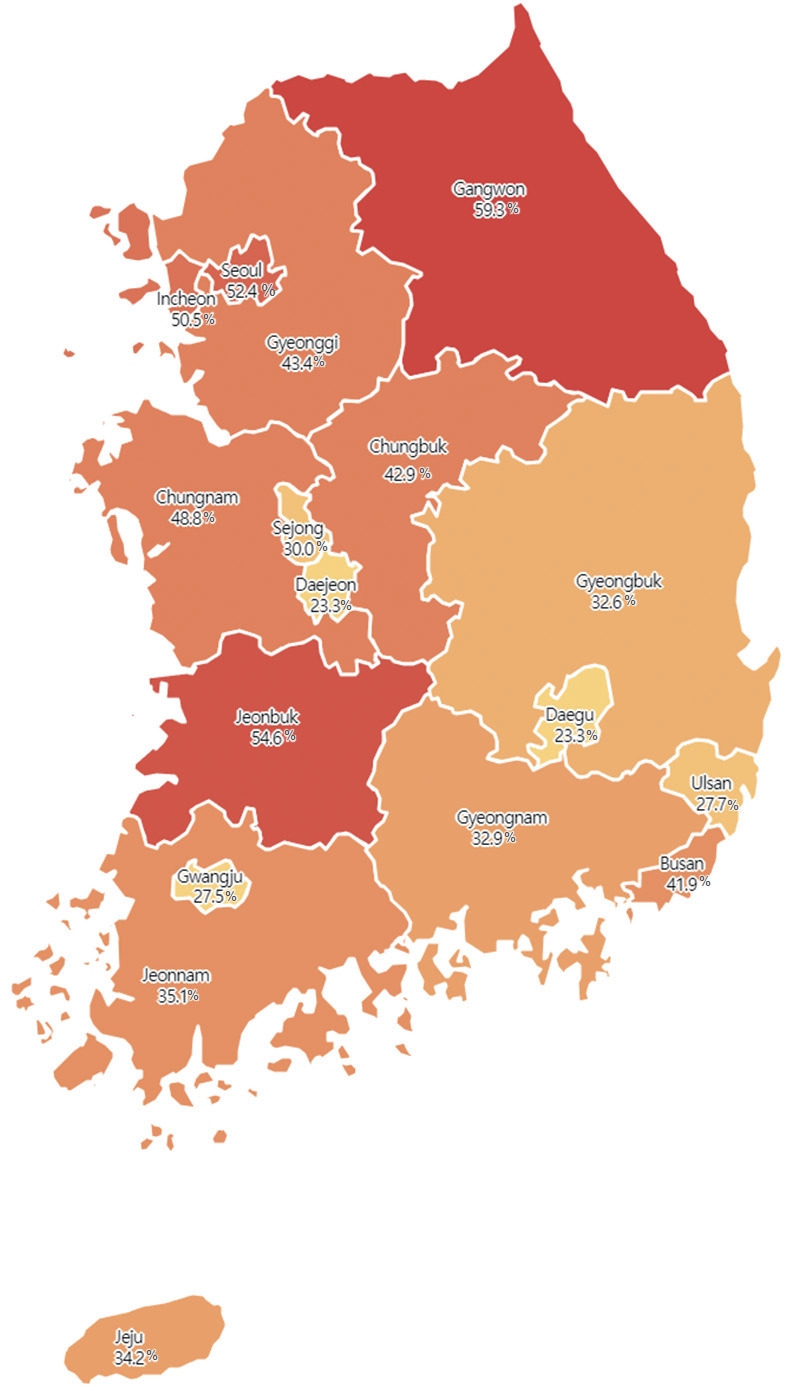
The palivizumab coverage rate observed in residential areas. The color gradient (yellow to red) indicates the palivizumab coverage rate, with red representing higher coverage rates and yellow representing lower coverage rates, highlighting regional differences in palivizumab administration.

Table 1 presents the baseline characteristics of the study population eligible for palivizumab administration. Infants with higher GA had lower coverage rates compared to those with lower GA. The non-palivizumab group had significantly lower rates of low birth weight (31.4 vs. 65.4%, p<0.001), NICU admission (32.0 vs. 86.2%, p<0.001), and perinatal morbidities—including respiratory distress syndrome, sepsis, necrotizing enterocolitis, intraventricular hemorrhage, and ROP—than the palivizumab group. Among the birth months, March had the lowest coverage rate at 27.8%. Additionally, coverage rates varied significantly by residential area. A higher proportion of infants who received palivizumab resided in Seoul and metropolitan areas, including Incheon and Gyeonggi-do (55.8%), whereas infants in the non-palivizumab group were more likely to reside in non-capital areas (53.6%, p<0.001; Figure 3). The proportion of mothers younger than 35 years was also higher in the non-palivizumab group compared to the palivizumab group (52.5 vs. 47.1%, p=0.005). In the multivariable logistic regression analysis, factors independently associated with non-administration of palivizumab included higher GA, female sex, non-low birth weight, birth in March, residence in non-capital areas, absence of NICU admission at birth, absence of perinatal comorbidities such as ROP, and maternal age less than 35 years (Table 2).

A subgroup analysis based on GA was performed due to the significant difference in coverage rates between infants born at 32-34 weeks GA and those born at 35 weeks GA. Detailed baseline characteristics by GA (32-34 vs. 35 weeks) are presented in Supplementary Materials 5 and 6. In subgroup analyses, factors independently associated with non-administration of palivizumab in infants born at 35 weeks GA included female sex, non-low birth weight, birth in March, residence in non-capital areas, absence of NICU admission at birth, absence of perinatal comorbidities such as RDS, and current maternal smoking status. For infants born at 32-34 weeks GA, factors associated with non-administration were similar to those in the 35 weeks GA group, including non-low birth weight, birth in March, residence in non-capital areas, medical aid insurance, absence of NICU admission at birth, and absence of perinatal comorbidities such as ROP (Supplementary Material 7).

## DISCUSSION

In this study, we evaluated the coverage rates of palivizumab in MLPT infants following the implementation of palivizumab prophylaxis. We identified factors influencing these coverage rates using the Korean NHIS database. Between October 2016 and March 2019, the overall coverage rate was 42.2%, with rates increasing over time. However, infants with higher GAs, females, those born in March, residents of metropolitan and rural areas outside Seoul, and infants whose mothers were under 35 years had significantly lower coverage rates. Conversely, no statistically significant correlation was found between insurance type, socioeconomic status, and the coverage rate.

When analyzing the coverage rates among MLPT infants eligible for palivizumab reimbursement, we observed that the rate in the first year after reimbursement was introduced was 37.8%. Subsequently, the coverage rate remained consistently low, staying below 50% even after 2 years. Compliance with palivizumab prophylaxis ranges from 25% to 100%, depending on the population and healthcare system [17]. Few studies have specifically examined the coverage rate of palivizumab in the MLPT population because guidelines regarding its administration vary across countries. In a study from the United Arab Emirates, MLPT infants aged ≤6 months at the onset of the RSV season with an RSV high-risk score of ≥49 were indicated for palivizumab prophylaxis, and compliance was reported at 91% [18]. The study suggested that this high compliance might be directly associated with increased awareness campaigns and focused caregiver approaches [18]. Monitoring coverage rates and compliance is essential for developing effective strategies to enhance prophylaxis and prevent severe RSV infections.

In our study, an increasing trend in palivizumab coverage rates was observed as the seasons progressed, a trend also noted in other studies [18,19]. When stratified by GA at birth, seasonal changes in coverage rates varied by GA. Infants born at 32 weeks GA consistently had high coverage rates—nearly 80% from the first season—with little variation thereafter. In contrast, infants born at 35 weeks GA had coverage rates below 50% in the first season, followed by a gradual and significant increase in later seasons. This distinct pattern warrants further analysis and discussion. Beyond seasonal trends, notable differences were evident between GA groups; infants born at 32-34 weeks GA generally exhibited higher palivizumab coverage than those born at 35 weeks GA. Several factors may contribute to this phenomenon. First, these patterns may reflect differences in the medical conditions of infants born at 32-34 weeks GA versus those born at 35 weeks GA. Moderate preterm infants are admitted to the NICU at higher rates than late preterm infants [20]. They tend to experience more respiratory, gastrointestinal, and feeding problems, as well as ROP, during the early postnatal period compared to late preterm infants [21-23]. Furthermore, even after discharge, moderate preterm infants are at a higher risk of readmission, in-hospital mortality [20], home oxygen use [24], and long-term neurodevelopmental impairments than late preterm infants [25,26]. Among MLPT infants, those born at younger GAs are more likely to experience severe outcomes related to RSV infection—such as hospitalization, ICU admissions, and mechanical ventilation—which result in higher hospital charges [27,28]. Thus, the increased severity of illness and higher healthcare utilization in infants with lower GA may raise awareness among healthcare professionals and caregivers, potentially leading to higher palivizumab coverage. Second, parents of infants born at 35 weeks GA may perceive their babies as less vulnerable than those born earlier, resulting in decreased awareness of RSV risks and lower uptake of preventive measures like palivizumab. Third, the national reimbursement program in Korea covers infants born before 35 weeks and 6 days of gestation. Some healthcare providers may misunderstand or be unclear about this cutoff, leading to the under-prescription of palivizumab for infants born at 35 weeks GA. Therefore, improved education for both patients and healthcare providers, along with tailored approaches, is necessary to enhance coverage in this population.

In our study, palivizumab coverage rates varied by birth month, with the highest rate observed in December and the lowest in March. This pattern is likely associated with the seasonal occurrence of RSV. Analysis of weekly hospitalized RSV cases in children under 6 years old, based on sentinel surveillance by the Korea Disease Control and Prevention Agency, revealed a consistent annual pattern [29]. For instance, 2017-2018 data showed that case numbers gradually increased to approximately 500-700 per month in October, peaked at over 2,000 per month in November and December, and then declined [29]. These monthly variations in RSV occurrence may have influenced both awareness of the risks and the perceived need for prophylaxis, thereby contributing to differences in palivizumab coverage rates.

This study also observed significant regional disparities in palivizumab coverage rates. These disparities may be related to an unequal allocation of healthcare resources across regions. For example, the higher NICU admission rate in the administration group suggests that palivizumab was primarily administered in tertiary or general hospitals with NICU facilities. According to the “Analysis of the Trends in the Healthcare Services Industry in 2017” report, 32.6% of tertiary general hospitals are located in Seoul, with an additional 18.6% in the Incheon/Gyeonggi region [30]. Moreover, 14.1% of general hospitals are in Seoul and 23.8% in the Incheon/Gyeonggi region. A 2016 report on the “Evaluation of Performance and Efficiency in Operation of Neonatal Intensive Care Unit” noted that 56.2% of NICUs nationwide were concentrated in Seoul and the Incheon/Gyeonggi region [31]. In addition to disparities in facilities, there was also an imbalance in medical personnel, with 61.4% of neonatal specialists concentrated in the Seoul and Incheon/Gyeonggi regions [31]. Despite these observations, the regional disparities in palivizumab coverage rates cannot be fully explained by NICU facilities, personnel, and healthcare resources alone. Figure 3 shows patterns that challenge this reasoning, such as high coverage rates in Gangwon and Jeonbuk alongside low rates in the 5 major metropolitan cities. In addition to resource allocation, local healthcare policies, differences in provider awareness or education regarding palivizumab, and variations in parental attitudes or socioeconomic factors may also contribute to these regional differences. Further investigation into these aspects is needed for a more comprehensive explanation.

In our study, neither insurance type nor socioeconomic status significantly influenced palivizumab coverage rates. Palivizumab is an expensive medication, costing approximately 500,000 Korean won for a 50 mg vial and about 900,000 Korean won for a 100 mg vial. Moreover, because dosage increases with body weight, the cost rises with each additional dose. Nonetheless, socioeconomic status did not significantly affect coverage rates, possibly due to the expanded reimbursement system and national support.

Several studies have evaluated the effectiveness of palivizumab in MLPT infants. Under the 2009 American Academy of Pediatrics policy, the maximum number of palivizumab doses for preterm infants born between 32 0/7 weeks and 34 6/7 weeks GA was reduced from 5 to 3. Additionally, the criteria for qualifying risk factors were narrowed, resulting in fewer infants being eligible for palivizumab prophylaxis and, among those eligible, fewer doses being administered. These policy changes were associated with an increased incidence of recurrent wheezing up to 12 months of age, adjusted for prematurity [32]. In Quebec, Canada, before the 2015-2016 season, preterm infants born at 33-35 weeks GA without other qualifying comorbidities were eligible for immunoprophylaxis if they were under 6 months old at the start of, or born during, the RSV season. According to the Canadian risk scoring tool for RSV hospitalization, these infants were classified as moderate to high risk. However, in 2015 the Quebec Ministry of Health revised the immunoprophylaxis program so that infants without additional qualifying comorbidities were no longer eligible. A comparison of outcomes before and after this change revealed that the withdrawal of palivizumab prophylaxis significantly increased RSV-associated ALRI hospitalizations by age 2 [33]. In Korea, we observed that following the 2016 insurance implementation of palivizumab prophylaxis for MLPT infants born during the RSV season with older siblings, the incidence of severe ALRI-related hospitalizations decreased significantly from 26.0% in the pre-insurance period to 24.0% in the insurance period (p<0.05) [34]. This reduction in severe ALRI risk was observed only in infants born during the RSV season (decreasing from 26.4 to 22.7%, p<0.01), with no significant change in those born during the non-RSV season (25.6 to 25.7%) [34].

The direct impact of palivizumab on RSV-related disease is further underscored by its cost-effectiveness. Previous studies have demonstrated that palivizumab offers both direct and indirect cost benefits by preventing severe RSV-related ALRI in MLPT infants, particularly in those with more than 2 risk factors or at least a moderate risk level according to a risk scoring tool [35,36]. Further cost-effectiveness studies evaluating the economic implications of palivizumab use in Korean MLPT infants would provide valuable insights for pediatricians making informed decisions regarding resource allocation and RSV prevention strategies.

This study has several limitations. First, because it evaluated palivizumab coverage rates using Korea’s nationwide database, the findings may not be generalizable to other countries given Korea’s unique regional, medical, and epidemiological characteristics. Second, our analysis covered 3 RSV seasons following the extension of palivizumab coverage for MLPT infants—a period that may have coincided with inadequate education and promotion of palivizumab prophylaxis. Additional analyses are needed to explore factors influencing coverage rates once administration stabilizes after sufficient education and promotion. Third, regional coverage rates were analyzed based on the participants’ places of residence rather than the locations of healthcare facilities, which may not accurately reflect coverage within specific institutions. Fourth, the dataset did not include information on the age of older siblings, precluding analysis of the potential influence of siblings’ ages on palivizumab coverage rates.

While our study underscores the importance of palivizumab prophylaxis in MLPT infants, the evolving landscape of RSV prevention guidelines must be acknowledged. The AAP removed MLPT infants as an eligible category for palivizumab prophylaxis in 2014 [37]. Furthermore, several European countries have narrowed the indications for palivizumab use [38]. These changes reflect the ongoing debate and evolving understanding of the risk–benefit balance of palivizumab in this population. Our findings should be interpreted in the context of these international trends, and their potential implications for future RSV prevention strategies in Korea should be considered.

In Korea, following the introduction of palivizumab for MLPT infants, the overall coverage rate during the initial 3 RSV seasons was low (42.2%). Infants with higher gestational ages, those born in March, and residents of non-capital areas exhibited significantly lower coverage rates. These findings provide valuable insights for government and health authorities, aiding in the formulation and implementation of policies to improve palivizumab coverage and enhance overall infant health against RSV infection.

## Figures and Tables

**Figure f4-epih-47-e2025015:**
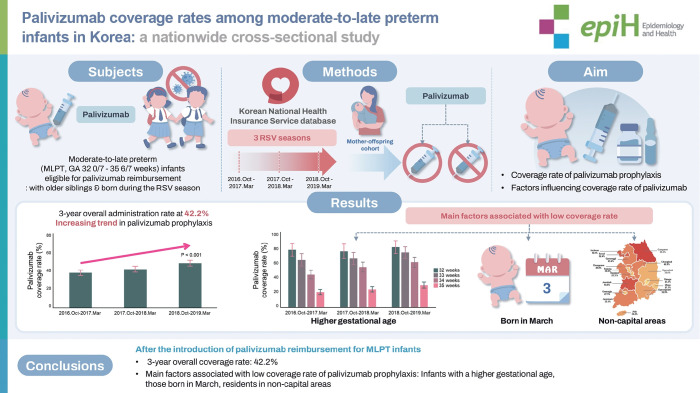


**Table 1.** Baseline characteristics of the study population eligible for palivizumab administration (n=2,843)

Characteristics	Non-palivizumab administration (n=1,642)	Palivizumab administration (n=1,201)	Coverage rate (%)	p-value
Infant factors				
Gestational age (wk)	34.6±0.8	33.8±1.1	-	<0.001
32	50 (3.1)	187 (15.6)	78.9	<0.001
33	118 (7.2)	255 (21.2)	68.4	
34	334 (20.3)	376 (31.3)	53.0	
35	1,140 (69.4)	383 (31.9)	25.1	
Male sex	879 (53.5)	686 (57.1)	43.8	0.058
SGA	20 (1.2)	25 (2.1)	55.6	0.068
LGA	7 (0.4)	4 (0.3)	36.4	0.692
LBW	516 (31.4)	785 (65.4)	60.3	<0.001
Multiple birth	358 (21.8)	280 (23.3)	43.9	0.340
Birth month				<0.001
Oct	271 (16.5)	192 (16.0)	41.5	
Nov	253 (15.4)	201 (16.7)	44.3	
Dec	203 (12.4)	216 (18.0)	51.6	
Jan	299 (18.2)	243 (20.2)	44.8	
Feb	229 (13.9)	200 (16.7)	46.6	
Mar	387 (23.6)	149 (12.4)	27.8	
Residential area				<0.001
Seoul	180 (11.0)	198 (16.5)	52.4	
Incheon-Gyeonggi	582 (35.4)	472 (39.3)	44.8	
Non-capital areas	880 (53.6)	531 (44.2)	37.6	
NICU admission	526 (32.0)	1,035 (86.2)	66.3	<0.001
Comorbidities				
RDS	352 (21.4)	466 (38.8)	57.0	<0.001
Sepsis	39 (2.4)	60 (5.0)	60.6	<0.001
NEC	2 (0.1)	10 (0.8)	83.3	0.004
IVH	22 (1.3)	36 (3.0)	62.1	0.002
ROP	11 (0.7)	38 (3.2)	77.6	<0.001
RSV season year				<0.001
Oct 2016-Mar 2017	625 (38.1)	380 (31.6)	37.8	
Oct 2017-Mar 2018	552 (33.6)	393 (32.7)	41.6	
Oct 2018-Mar 2019	465 (28.3)	428 (35.7)	47.9	
Maternal factors				
Maternal age (yr)	34.0±4.1	34.5±3.9		0.005
<35	862 (52.5)	566 (47.1)	39.6	0.005
≥35	780 (47.5)	635 (52.9)	44.9	
BMI (kg/m^2^)	21.8±3.6	22.1±3.8		0.088
Insurance type				0.163
Medical insurance	1,619 (98.6)	1,191 (99.2)	42.4	
Medical Aid	23 (1.4)	10 (0.8)	30.3	
Socioeconomic status				0.092
Low income	290/1,642 (17.7)	215/1,199 (17.9)	42.6	
Middle income	662/1,642 (40.3)	437/1,199 (36.5)	39.8	
High income	690/1,642 (42.0)	547/1,199 (45.6)	44.2	
Smoking				0.115
Never	921/1,063 (86.6)	711/795 (89.4)	43.6	
Former	56/1,063 (5.3)	39/795 (4.9)	41.1	
Current	86/1,063 (8.1)	45/795 (5.7)	34.4	
Drinking				0.545
<2-3 times/mo	207/788 (26.3)	157/555 (28.3)	43.1	
<1-2 times/wk	224/788 (28.4)	168/555 (30.3)	42.9	
3-4 times/wk	137/788 (17.4)	92/555 (16.6)	40.2	
Almost everyday	220/788 (27.9)	138/555 (24.9)	38.5	

Values are presented as mean±standard deviation or number (%). SGA, small-for-gestational-age; LGA, light-for-gestational age; LBW, low birth weight; NICU, neonatal intensive care unit; RDS, respiratory distress syndrome; NEC, necrotizing enterocolitis; IVH, intraventricular hemorrhage; ROP, retinopathy of prematurity; RSV, respiratory syncytial virus; BMI, body mass index.

**Table 2.** Predictive factors for the palivizumab administration in moderate-to-late preterm infants

Characteristics	Multivariable analysis^1^	p-value
GA (wk)		<0.001
32	1.00 (reference)	
33	0.63 (0.41, 0.97)	
34	0.44 (0.29, 0.65)	
35	0.27 (0.18, 0.39)	
Sex		0.046
Male	1.00 (reference)	
Female	0.82 (0.68, 0.99)	
LBW		<0.001
Yes	1.00 (reference)	
No	0.55 (0.45, 0.68)	
Birth month		<0.001
Oct	1.00 (reference)	
Nov	1.19 (0.85, 1.64)	
Dec	1.50 (1.07, 2.10)	
Jan	1.15 (0.84, 1.58)	
Feb	1.25 (0.89, 1.75)	
Mar	0.48 (0.34, 0.66)	
Residential area		<0.001
Seoul	1.00 (reference)	
Incheon-Gyeonggi	0.66 (0.49, 0.89)	
Non-capital areas	0.44 (0.33, 0.60)	
Insurance type		0.157
Medical insurance	1.00 (reference)	
Medical Aid	0.51 (0.20, 1.29)	
NICU admission		<0.001
Yes	1.00 (reference)	
No	0.11 (0.09, 0.14)	
Comorbidities		
RDS	1.07 (0.86, 1.32)	0.520
Sepsis	0.85 (0.53, 1.35)	0.502
NEC	2.05 (0.40, 10.42)	0.385
IVH	0.89 (0.49, 1.61)	0.714
ROP	3.68 (1.61, 8.41)	0.002
Maternal age (yr)		0.005
≥35	1.00 (reference)	
<35	0.76 (0.62, 0.91)	

Values are presented as adjusted odds ratio (95% confidence interval). GA, gestational age; LBW, low birth weight;. NICU, neonatal intensive care unit; RDS, respiratory distress syndrome; NEC, necrotizing enterocolitis; IVH, intraventricular hemorrhage; ROP, retinopathy of prematurity.

1 Factors associated with palivizumab prophylaxis were identified using univariate analysis, which showed statistical significance; Adjusted factors included sex, GA, birth month and year, baseline diseases including RDS, sepsis, NEC, ROP, and IVH, LBW, residential area, NICU admission, and maternal factors (age and type of insurance).

## Supplementary materials

Supplementary Material 1. List of ICD-10 codes for exclusion diseases.

Supplementary Material 2. List of ICD-10 codes for baseline diseases.

Supplementary Material 3. Yearly trend of palivizumab coverage rate

Supplementary Material 4. Yearly trend of palivizumab coverage rate by gestational age groups 35 weeks and 32-34 weeks.

Supplementary Material 5. Baseline characteristics of the study population born at 32-34 weeks of gestation (n = 1,320).

Supplementary Material 6. Baseline characteristics of the study population born at 35 weeks of gestation (n = 1,523).

Supplementary Material 7. Predictive factors for palivizumab administration in preterm infants born at 35 weeks and 32-34 weeks of gestational age.

## References

[1] Li Y, Wang X, Blau DM, Caballero MT, Feikin DR, Gill CJ (2022). Global, regional, and national disease burden estimates of acute lower respiratory infections due to respiratory syncytial virus in children younger than 5 years in 2019: a systematic analysis. Lancet.

[2] Esposito S, Abu Raya B, Baraldi E, Flanagan K, Martinon Torres F, Tsolia M (2022). RSV prevention in all infants: which is the most preferable strategy?. Front Immunol.

[3] Englund JA, Cohen RA, Bianco V, Domachowske JB, Langley JM, Madhi SA (2023). Evaluation of clinical case definitions for respiratory syncytial virus lower respiratory tract infection in young children. J Pediatric Infect Dis Soc.

[4] Coutts J, Fullarton J, Morris C, Grubb E, Buchan S, Rodgers-Gray B (2020). Association between respiratory syncytial virus hospitalization in infancy and childhood asthma. Pediatr Pulmonol.

[5] Fauroux B, Simões EA, Checchia PA, Paes B, Figueras-Aloy J, Manzoni P (2017). The burden and long-term respiratory morbidity associated with respiratory syncytial virus infection in early childhood. Infect Dis Ther.

[6] Shi T, Ooi Y, Zaw EM, Utjesanovic N, Campbell H, Cunningham S (2020). Association between respiratory syncytial virus-associated acute lower respiratory infection in early life and recurrent wheeze and asthma in later childhood. J Infect Dis.

[7] Lanari M, Anderson EJ, Sheridan-Pereira M, Carbonell-Estrany X, Paes B, Rodgers-Gray BS (2020). Burden of respiratory syncytial virus hospitalisation among infants born at 32-35 weeks’ gestational age in the Northern Hemisphere: pooled analysis of seven studies. Epidemiol Infect.

[8] Greenberg D, Dagan R, Shany E, Ben-Shimol S, Givon-Lavi N (2020). Incidence of respiratory syncytial virus bronchiolitis in hospitalized infants born at 33-36 weeks of gestational age compared with those born at term: a retrospective cohort study. Clin Microbiol Infect.

[9] Greenberg D, Dagan R, Shany E, Bar-Ziv J, Givon-Lavi N (2014). Increased risk for respiratory syncytial virus-associated, community-acquired alveolar pneumonia in infants born at 31-36 weeks of gestation. Pediatr Infect Dis J.

[10] Figueras-Aloy J, Manzoni P, Paes B, Simões EA, Bont L, Checchia PA (2016). Defining the risk and associated morbidity and mortality of severe respiratory syncytial virus infection among preterm infants without chronic lung disease or congenital heart disease. Infect Dis Ther.

[11] Korean Statistical Information Service (2022 [cited 2024 Jan 14]). Population trends survey by province/gestational age. https://kosis.kr/statHtml/statHtml.do?sso=ok&returnurl=https%3A%2F%2Fkosis.kr%3A443%2FstatHtml%2FstatHtml.do%3Fconn_path%3DI2%26tblId%3DDT_1B81A15%26orgId%3D101%26.

[12] Seong SC, Kim YY, Khang YH, Park JH, Kang HJ, Lee H (2017). Data resource profile: the National Health Information Database of the National Health Insurance Service in South Korea. Int J Epidemiol.

[13] https://nhiss.nhis.or.kr/bd/ab/bdaba000eng.do.

[14] Choi EY, Jeong HE, Noh Y, Choi A, Yon DK, Han JY (2023). Neonatal and maternal adverse outcomes and exposure to nonsteroidal anti-inflammatory drugs during early pregnancy in South Korea: a nationwide cohort study. PLoS Med.

[15] Noh Y, Lee H, Choi A, Kwon JS, Choe SA, Chae J (2022). First-trimester exposure to benzodiazepines and risk of congenital malformations in offspring: a population-based cohort study in South Korea. PLoS Med.

[16] Kim HJ, Shah SC, Hann HJ, Kazmi SZ, Kang T, Lee JH (2021). Familial risk of inflammatory bowel disease: a population-based cohort study in South Korea. Clin Gastroenterol Hepatol.

[17] Frogel MP, Stewart DL, Hoopes M, Fernandes AW, Mahadevia PJ (2010). A systematic review of compliance with palivizumab administration for RSV immunoprophylaxis. J Manag Care Pharm.

[18] Elhalik M, El-Atawi K, Dash SK, Faquih A, Satyan AD, Gourshettiwar N (2019). Palivizumab prophylaxis among infants at increased risk of hospitalization due to respiratory syncytial virus infection in UAE: a hospital-based study. Can Respir J.

[19] Abushahin A, Janahi I, Tuffaha A (2018). Effectiveness of palivizumab immunoprophylaxis in preterm infants against respiratory syncytial virus disease in Qatar. Int J Gen Med.

[20] Iacobelli S, Combier E, Roussot A, Cottenet J, Gouyon JB, Quantin C (2017). Gestational age and 1-year hospital admission or mortality: a nation-wide population-based study. BMC Pediatr.

[21] Scheuchenegger A, Windisch B, Pansy J, Resch B (2023). Morbidities and rehospitalizations during the first year of life in moderate and late preterm infants: more similarities than differences?. Minerva Pediatr (Torino).

[22] Çömez A, Çelemler P, Özmen MC, Yurttutan S, Akkececi NS, Güngör K (2022). Retinopathy of prematurity incidence and treatment modalities in moderate and late preterm infants: a study from two tertiary centers. Can J Ophthalmol.

[23] Nair J, Longendyke R, Lakshminrusimha S (2018). Necrotizing enterocolitis in moderate preterm infants. Biomed Res Int.

[24] Lagatta JM, Clark RH, Brousseau DC, Hoffmann RG, Spitzer AR (2013). Varying patterns of home oxygen use in infants at 23-43 weeks’ gestation discharged from United States neonatal intensive care units. J Pediatr.

[25] Chyi LJ, Lee HC, Hintz SR, Gould JB, Sutcliffe TL (2008). School outcomes of late preterm infants: special needs and challenges for infants born at 32 to 36 weeks gestation. J Pediatr.

[26] Hirata K, Ueda K, Wada K, Ikehara S, Tanigawa K, Kimura T (2024). Neurodevelopmental outcomes at age 3 years after moderate preterm, late preterm and early term birth: the Japan Environment and Children’s Study. Arch Dis Child Fetal Neonatal Ed.

[27] Krilov LR, Forbes ML, Goldstein M, Wadhawan R, Stewart DL (2021). Severity and cost of RSV hospitalization among US preterm infants following the 2014 American Academy of Pediatrics policy change. Infect Dis Ther.

[28] Kong AM, Winer IH, Zimmerman NM, Diakun D, Bloomfield A, Gonzales T (2023). Increasing rates of RSV hospitalization among preterm infants: a decade of data. Am J Perinatol.

[29] https://dportal.kdca.go.kr/pot/is/st/ari.do.

[30] Park J (2017). Analysis of the trends in the healthcare services industry in 2017.

[31] Kim H (2016). Evaluation of performance and efficiency in operation of neonatal intensive care unit.

[32] Olicker A, Li H, Tatsuoka C, Ross K, Trembath A, Hibbs AM (2016). Have changing palivizumab administration policies led to more respiratory morbidity in infants born at 32-35 weeks?. J Pediatr.

[33] Papenburg J, Defoy I, Massé E, Caouette G, Lebel MH (2021). Impact of the withdrawal of palivizumab immunoprophylaxis on the incidence of respiratory syncytial virus (RSV) hospitalizations among infants born at 33 to 35 weeks’ gestational age in the province of Quebec, Canada: the RSV-Quebec Study. J Pediatric Infect Dis Soc.

[34] Kim S, Choe YJ, Lee S, Heo JS (2024). Impact of palivizumab in preventing severe acute lower respiratory infection in moderate-to-late preterm infants: a nationwide cohort study. J Korean Med Sci.

[35] Papenburg J, Saleem M, Teselink J, Li A, Caouette G, Massé É (2020). Cost-analysis of withdrawing immunoprophylaxis for respiratory syncytial virus in infants born at 33-35 weeks gestational age in Quebec: a multicenter retrospective study. Pediatr Infect Dis J.

[36] Lanctôt KL, Masoud ST, Paes BA, Tarride JE, Chiu A, Hui C (2008). The cost-effectiveness of palivizumab for respiratory syncytial virus prophylaxis in premature infants with a gestational age of 32-35 weeks: a Canadian-based analysis. Curr Med Res Opin.

[37] American Academy of Pediatrics Committee on Infectious Diseases, American Academy of Pediatrics Bronchiolitis Guidelines Committee (2014). Updated guidance for palivizumab prophylaxis among infants and young children at increased risk of hospitalization for respiratory syncytial virus infection. Pediatrics.

[38] Reeves RM, van Wijhe M, Lehtonen T, Stona L, Teirlinck AC, Vazquez Fernandez L (2022). A systematic review of European clinical practice guidelines for respiratory syncytial virus prophylaxis. J Infect Dis.

