# Supplementary material for: Palivizumab coverage rates among moderate-to-late preterm infants in Korea: a nationwide cross-sectional study
Source: Epidemiol Health. 2025 Apr 1;47:e2025015. doi: 10.4178/epih.e2025015 (PMC12178765; doi:10.4178/epih.e2025015)
Supplement: Supplementary Material 1. — List of ICD-10 codes for exclusion diseases. [file epih-47-e2025015-Supplementary-1.docx]

**Supplementary Material 1.** List of ICD-10 codes for exclusion diseases.

| **Disease** | **ICD-10 Codes** |
| --- | --- |
| Balanced rearrangements and structural markers, NEC | Q95 |
| Bronchopulmonary dysplasia | P27, P27.1, P27.8, P27.9, P27.10-P27.12, P27.19 |
| Congenital heart disease | Q20-28 (exclude Q25.0(PDA), Q21.12(PFO)), I50.0, P29.0, I27.0, P29.3 |
| Congenital malformations of larynx | Q31 |
| Congenital malformations of lung | Q33 |
| Congenital malformations of nose | Q30 |
| Congenital malformations of trachea and bronchus | Q32 |
| Combined immunodeficiencies | D81 |
| Common variable immunodeficiency | D83 |
| Cystic fibrosis | E84 |
| Disorders of muscle tone of newborn | P94 |
| Disorders of myoneural junction and muscle in diseases classified elsewhere | G73 |
| Down’s syndrome | Q90 |
| Edward’s syndrome and Patau’s syndrome | Q91 |
| Hypoxic ischemic encephalopathy of newborn | P91.6 |
| Immunodeficiency associated with other major defects | D82 |
| Immunodeficiency with predominantly antibody defects | D80 |
| Meconium ileus in cystic fibrosis | P75 |
| Monosomies and deletions from the autosomes, NEC | Q93 |
| Myasthenia gravis and other myoneural disorders | G70 |
| Neonatal cerebral leukomalacia | P91.2 |
| Other chromosome abnormalities, NEC | Q99 |
| Other congenital malformations of respiratory system | Q34 |
| Other immunodeficiencies | D84 |
| Other sex chromosome abnormalities, female phenotype, NEC | Q97 |
| Other sex chromosome abnormalities, male phenotype, NEC | Q98 |
| Other trisomies and partial trisomies of the autosomes, NEC | Q92 |
| Spinal muscular atrophy and related syndromes | G12 |
| Transplanted organ and tissue status | Z94 |
| Turner’s syndrome | Q96 |

ICD-10, International Classification of Disease 10^th^ Revision.
