# Supplementary material for: Palivizumab coverage rates among moderate-to-late preterm infants in Korea: a nationwide cross-sectional study
Source: Epidemiol Health. 2025 Apr 1;47:e2025015. doi: 10.4178/epih.e2025015 (PMC12178765; doi:10.4178/epih.e2025015)
Supplement: Supplementary Material 2. — List of ICD-10 codes for baseline diseases. [file epih-47-e2025015-Supplementary-2.docx]

**Supplementary Material 2.** List of ICD-10 codes for baseline diseases.

| **Disease** | **ICD-10 Codes** |
| --- | --- |
| Intraventricular hemorrhage | I61.5, P10.2, P52.0-P52.3 |
| Small-for-gestational age | P051 |
| Light-for-gestational age | P050 |
| Low birth weight | P07, P07.0, P07.1 |
| Necrotizing enterocolitis | P77 |
| Respiratory distress syndrome | P22, P22.0, P22.1, P22.8, P22.9 |
| Retinopathy of prematurity | H35.1 |
| Sepsis | A02.1, A20.7, A22.7, A24.1, A26.7, A32.7, A40, A40.0-A40.3, A40.8, A40.9, A41, A41.0-A41.5, A41.50-A41.53, A41.58, A41.59, A41.8, A41.80, A41.88, A41.9, A42.7, A54.8, B00.7, B37.7, J95.0, P36, P36.0-P36.5, P36.8, P36.9, R65.1, T80.2, T81.4 |

ICD-10, International Classification of Disease 10^th^ Revision.
