# Supplementary figures and images for: Palivizumab coverage rates among moderate-to-late preterm infants in Korea: a nationwide cross-sectional study
Source: Epidemiol Health. 2025 Apr 1;47:e2025015. doi: 10.4178/epih.e2025015 (PMC12178765; doi:10.4178/epih.e2025015)

**Supplementary Material 3.** Yearly trend of palivizumab coverage rate


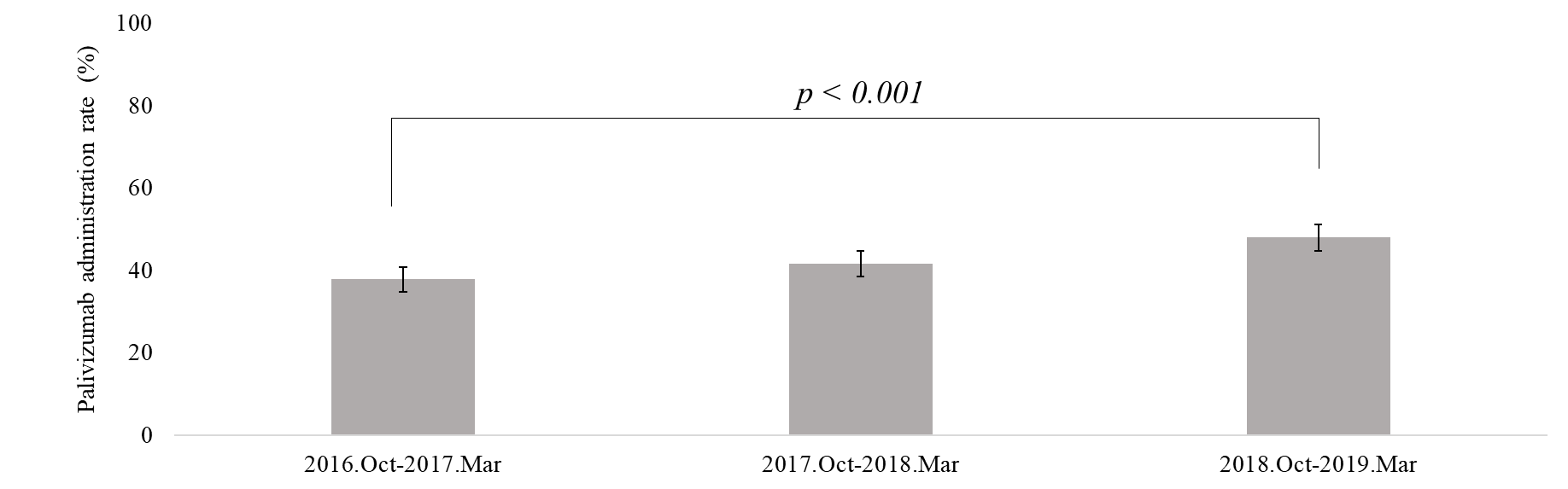

Supplement: Supplementary Material 3. — Yearly trend of palivizumab coverage rate [file epih-47-e2025015-Supplementary-3.docx]
