# Supplementary material for: Palivizumab coverage rates among moderate-to-late preterm infants in Korea: a nationwide cross-sectional study
Source: Epidemiol Health. 2025 Apr 1;47:e2025015. doi: 10.4178/epih.e2025015 (PMC12178765; doi:10.4178/epih.e2025015)
Supplement: Supplementary Material 4. — Yearly trend of palivizumab coverage rate by gestational age groups 35 weeks and 32-34 weeks. [file epih-47-e2025015-Supplementary-4.docx]

**Supplementary Material 4.** Yearly trend of palivizumab coverage rate by gestational age groups 35 weeks and 32-34 weeks.


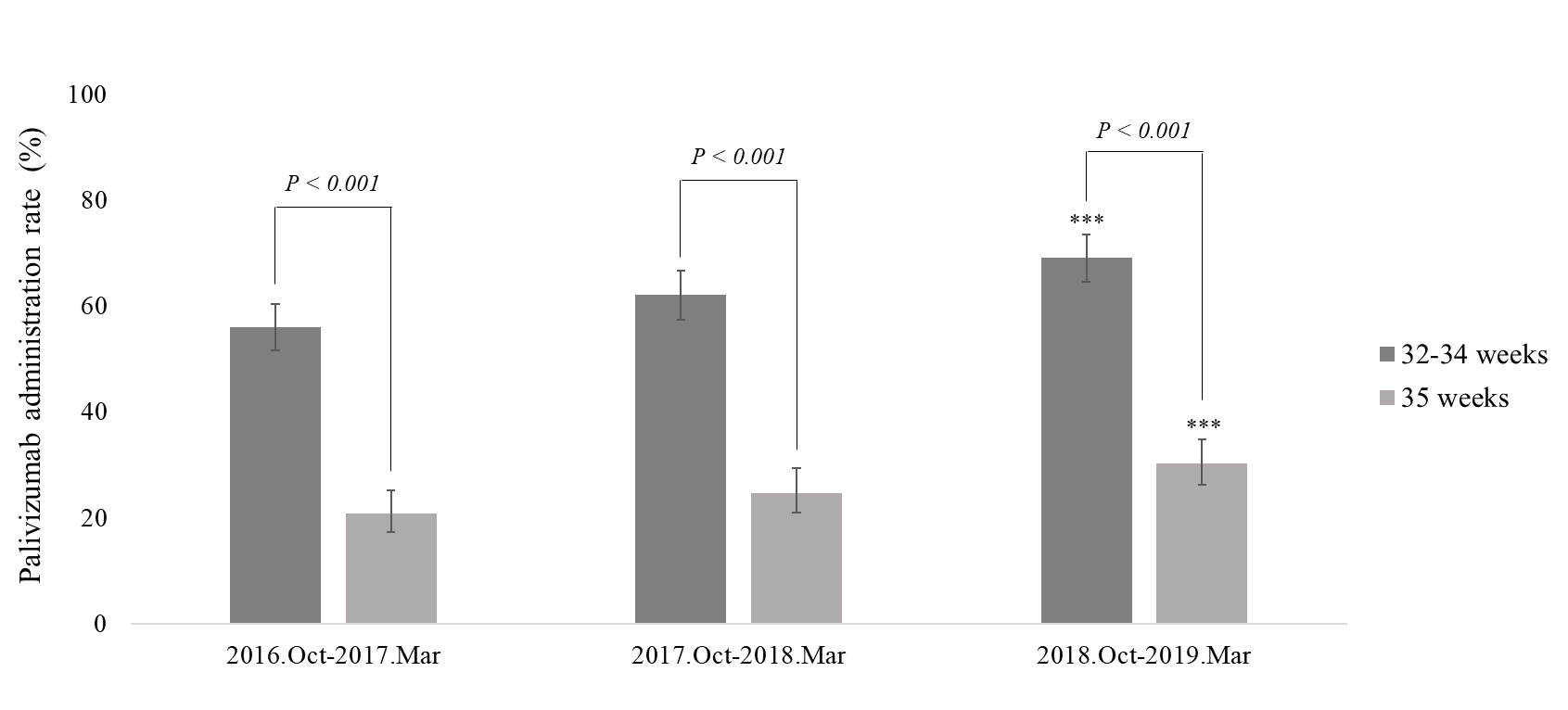


*** ***P*** < 0.001 compared to 2016 season
