# Supplementary material for: Palivizumab coverage rates among moderate-to-late preterm infants in Korea: a nationwide cross-sectional study
Source: Epidemiol Health. 2025 Apr 1;47:e2025015. doi: 10.4178/epih.e2025015 (PMC12178765; doi:10.4178/epih.e2025015)
Supplement: Supplementary Material 5. — Baseline characteristics of the study population born at 32-34 weeks of gestation (n = 1,320). [file epih-47-e2025015-Supplementary-5.docx]

Supplementary Material 5. Baseline characteristics of the study population born at 32-34 weeks of gestation (n = 1,320).

| **Characteristics** | **Non-palivizumab administration**  **(n = 502)** | **Palivizumab administration**  **(n = 818)** | ***P-*value** |
| --- | --- | --- | --- |
| **Infant factors** |  |  |  |
| Male sex | 283 (56.4) | 464 (56.7) | 0.901 |
| SGA | 4 (0.8) | 16 (2.0) | 0.094 |
| LGA | 2 (0.4) | 1 (0.1) | 0.561 |
| LBW | 268 (53.4) | 598 (73.1) | < 0.001 |
| Multiple birth | 110 (21.9) | 187 (22.9) | 0.689 |
| Birth Month |  |  |  |
| October | 81 (16.1) | 131 (16.0) | < 0.001 |
| November | 69 (13.8) | 138 (16.9) |  |
| December | 62 (12.4) | 151 (18.5) |  |
| January | 92 (18.3) | 172 (21.0) |  |
| February | 76 (15.1) | 131 (16.0) |  |
| March | 122 (24.3) | 95 (11.6) |  |
| Residential area |  |  |  |
| Seoul | 44 (8.7) | 117 (14.3) | < 0.001 |
| IncheonￚGyeonggi | 167 (33.3) | 331 (40.5) |  |
| Non-Capital Areas | 291 (58.0) | 370 (45.2) |  |
| NICU admission | 280 (55.8) | 768 (93.9) | < 0.001 |
| Comorbidities |  |  |  |
| RDS | 153 (30.5) | 338 (41.3) | < 0.001 |
| Sepsis | 20 (4.0) | 45 (5.5) | 0.216 |
| NEC | 1 (0.2) | 10 (1.2) | 0.060 |
| IVH | 11 (2.2) | 23 (2.8) | 0.490 |
| ROP | 6 (1.2) | 35 (4.3) | 0.002 |
| RSV season year |  |  |  |
| 2016.10–2017.03 | 214 (42.6) | 272 (33.3) | < 0.001 |
| 2017.10–2018.03 | 162 (32.3) | 265 (32.4) |  |
| 2018.10–2019.03 | 126 (25.1) | 281 (34.4) |  |
| **Maternal factors** |  |  |  |
| Maternal age (y) | 34.3 ± 3.9 | 34.5 ± 3.8 | 0.362 |
| < 35 y | 265 (52.8) | 395 (48.3) | 0.112 |
| ≥ 35 y | 237 (47.2) | 423 (51.7) |  |
| BMI (kg/m^2^) | 22.4 ± 4.0 | 22.1 ± 3.7 | 0.381 |
| Insurance type |  |  |  |
| Medical Insurance | 494 (98.4) | 813 (99.4) | 0.091 |
| Medical aid | 8 (1.6) | 5 (0.6) |  |
| Socioeconomic status |  |  |  |
| Low income | 85 (16.9) | 158/816 (19.4) | 0.468 |
| Middle income | 189 (37.7) | 292/816 (35.8) |  |
| High income | 228 (45.4) | 366/816 (44.9) |  |
| Smoking |  |  |  |
| Never | 293/339 (86.4) | 477/543 (87.8) | 0.354 |
| Former | 14/339 (4.1) | 28/543 (5.2) |  |
| Current | 32/339 (9.4) | 38/543 (7.0) |  |
| Drinking |  |  |  |
| < 2–3 times/mo | 74/253 (29.3) | 115/388 (29.6) | 0.990 |
| < 1–2 times/wk | 78/253 (30.8) | 117/388 (30.2) |  |
| 3–4 times/wk | 40/253 (15.8) | 59/388 (15.2) |  |
| Almost everyday | 61/253 (2431) | 97/388 (25.0) |  |

Values are presented as mean±standard deviation or number (%).

BMI, body mass index; IVH, intraventricular hemorrhage; LBW, low birth weight; LGA, light-for-gestational age; NEC, necrotizing enterocolitis; NICU, neonatal intensive care unit; RDS, respiratory distress syndrome; ROP, retinopathy of prematurity; SD, standard deviation; SGA, small-for-gestational-age; wks, weeks.
