# Supplementary material for: Palivizumab coverage rates among moderate-to-late preterm infants in Korea: a nationwide cross-sectional study
Source: Epidemiol Health. 2025 Apr 1;47:e2025015. doi: 10.4178/epih.e2025015 (PMC12178765; doi:10.4178/epih.e2025015)
Supplement: Supplementary Material 6. — Baseline characteristics of the study population born at 35 weeks of gestation (n = 1,523). [file epih-47-e2025015-Supplementary-6.docx]

Supplementary Material 6. Baseline characteristics of the study population born at 35 weeks of gestation (n = 1,523).

| **Characteristics** | **Non-palivizumab administration**  **(n = 1,140)** | **Palivizumab administration**  **(n = 383)** | ***P-*value** |
| --- | --- | --- | --- |
| **Infant factors** |  |  |  |
| Male sex | 596 (52.3) | 222 (58.0) | 0.054 |
| SGA | 16 (1.4) | 9 (2.4) | 0.207 |
| LGA | 5 (0.4) | 3 (0.8) | 0.423 |
| LBW | 248 (21.8) | 187 (48.8) | < 0.001 |
| Multiple birth | 248 (21.8) | 93 (24.3) | 0.305 |
| Birth Month |  |  |  |
| October | 190 (16.7) | 61 (15.9) | 0.001 |
| November | 184 (16.1) | 63 (16.5) |  |
| December | 141 (12.4) | 65 (17.0) |  |
| January | 207 (18.2) | 71 (18.5) |  |
| February | 153 (13.4) | 69 (18.0) |  |
| March | 265 (23.3) | 54 (14.1) |  |
| Residential area |  |  |  |
| Seoul | 136 (11.9) | 81 (21.2) | < 0.001 |
| IncheonￚGyeonggi | 415 (36.4) | 141 (36.8) |  |
| Non-Capital Areas | 589 (51.7) | 161 (42.0) |  |
| NICU admission | 246 (21.6) | 267 (69.7) | < 0.001 |
| Comorbidities |  |  |  |
| RDS | 199 (17.5) | 128 (33.4) | < 0.001 |
| Sepsis | 19 (1.7) | 15 (3.9) | 0.010 |
| NEC | 1 (0.1) | 0 (0.0) | - |
| IVH | 11 (1.0) | 13 (3.4) | 0.001 |
| ROP | 5 (0.4) | 3 (0.8) | 0.430 |
| RSV season year |  |  |  |
| 2016.10–2017.03 | 411 (36.1) | 108 (28.2) | 0.003 |
| 2017.10–2018.03 | 390 (34.2) | 128 (33.4) |  |
| 2018.10–2019.03 | 339 (29.7) | 147 (38.4) |  |
| **Maternal factors** |  |  |  |
| Maternal age (y) | 33.9 ± 4.1 | 34.5 ± 4.0 | 0.032 |
| < 35 y | 597 (52.4) | 171 (44.7) | 0.009 |
| ≥ 35 y | 543 (47.6) | 212 (55.4) |  |
| BMI (kg/m^2^) | 21.6 ± 3.4 | 22.1 ± 3.8 | 0.041 |
| Insurance type |  |  | 0.988 |
| Medical Insurance | 1,125 (98.7) | 378 (98.7) |  |
| Medical aid | 15 (1.3) | 5 (1.3) |  |
| Socioeconomic status |  |  |  |
| Low income | 205 (18.0) | 57 (14.9) | 0.061 |
| Middle income | 473 (41.5) | 145 (37.9) |  |
| High income | 462 (40.5) | 181 (47.3) |  |
| Smoking |  |  |  |
| Never | 628/724 (86.7) | 234/252 (92.9) | 0.018 |
| Former | 42/724 (5.8) | 11/252 (4.4) |  |
| Current | 54/724 (7.5) | 7/252 (2.8) |  |
| Drinking |  |  |  |
| < 2–3 times/mo | 133/535 (24.9) | 42/167 (25.2) | 0.602 |
| < 1–2 times/wk | 146/535 (27.3) | 51/167 (30.5) |  |
| 3–4 times/wk | 97/535 (18.1) | 33/167 (19.8) |  |
| Almost everyday | 159/535 (29.7) | 41/167 (24.6) |  |

Values are presented as mean±standard deviation or number (%).

BMI, body mass index; IVH, intraventricular hemorrhage; LBW, low birth weight; LGA, light-for-gestational age; NEC, necrotizing enterocolitis; NICU, neonatal intensive care unit; RDS, respiratory distress syndrome; ROP, retinopathy of prematurity; SD, standard deviation; SGA, small-for-gestational-age; wks, weeks.
