# Supplementary material for: Palivizumab coverage rates among moderate-to-late preterm infants in Korea: a nationwide cross-sectional study
Source: Epidemiol Health. 2025 Apr 1;47:e2025015. doi: 10.4178/epih.e2025015 (PMC12178765; doi:10.4178/epih.e2025015)
Supplement: Supplementary Material 7. — Predictive factors for palivizumab administration in preterm infants born at 35 weeks and 32-34 weeks of gestational age. [file epih-47-e2025015-Supplementary-7.docx]

**Supplementary Material 7.** Predictive factors for palivizumab administration in preterm infants born at 35 weeks and 32-34 weeks of gestational age.

| **Characteristics** | **35 weeks of gestational age^a^** | | **32-34 weeks of gestational age^b^** | |
| --- | --- | --- | --- | --- |
|  | **Adj OR (95% CI)** | ***P-*value** | **Adj OR (95% CI)** | ***P-*value** |
| Sex |  | 0.027 |  | 0.408 |
| Male | Ref |  | Ref |  |
| Female | 0.73 (0.55 - 0.97) |  | 0.89 (0.69 - 1.17) |  |
| LBW |  | < 0.001 |  | 0.008 |
| Yes | Ref |  | Ref |  |
| No | 0.40 (0.30 - 0.54) |  | 0.68 (0.51 - 0.91) |  |
| Birth Month |  | 0.008 |  | < 0.001 |
| October | Ref |  | Ref |  |
| November | 0.97 (0.60 - 1.56) |  | 1.38 (0.87 - 2.20) |  |
| December | 1.42 (0.88 - 2.31) |  | 1.68 (1.04 - 2.70) |  |
| January | 1.18 (0.74 - 1.87) |  | 1.13 (0.73 - 1.74) |  |
| February | 1.28 (0.80 - 2.07) |  | 1.15 (0.73 - 1.83) |  |
| March | 0.60 (0.37 - 0.96) |  | 0.40 (0.26 - 0.62) |  |
| Residential area |  | < 0.001 |  | < 0.001 |
| Seoul | Ref |  | Ref |  |
| IncheonￚGyeonggi | 0.58 (0.39 - 0.86) |  | 0.72 (0.45 - 1.15) |  |
| Non-Capital Areas | 0.43 (0.29 - 0.64) |  | 0.45 (0.29 - 0.70) |  |
| Insurance type |  | 0.744 |  | 0.013 |
| Medical insurance | Ref |  | Ref |  |
| Medical aid | 1.21 (0.39 - 3.79) |  | 0.19 (0.05 - 0.71) |  |
| NICU admission |  | < 0.001 |  | < 0.001 |
| Yes | Ref |  | Ref |  |
| No | 0.15 (0.11 - 0.20) |  | 0.08 (0.05 - 0.11) |  |
| Comorbidities |  |  |  |  |
| RDS | 1.44 (1.05 - 1.98) | 0.024 | 0.96 (0.73 - 1.27) | 0.766 |
| ROP | - |  | 6.76 (2.39 - 19.15) | < 0.001 |
| Smoking |  | 0.029 | - |  |
| Never | Ref |  |  |  |
| Former | 0.48 (0.22 - 1.08) |  |  |  |
| Current | 0.32 (0.13 - 0.79) |  |  |  |
| Maternal age |  | 0.080 | - |  |
| ≥ 35 y | Ref |  |  |  |
| < 35 y | 0.78 (0.59 - 1.03) |  |  |  |

^a^Factors associated with palivizumab prophylaxis were identified using univariate analysis, which showed statistical significance. Adjusted factors included sex, birth month and year, baseline diseases including respiratory distress syndrome, low birth weight, residential area, NICU admission, and maternal factors (age and smoking status).

^b^Factors associated with palivizumab prophylaxis were identified using univariate analysis, which showed statistical significance. Adjusted factors included sex, birth month and year, baseline diseases including respiratory distress syndrome, and retinopathy of prematurity, low birth weight, residential area, NICU admission, and maternal factors as type of insurance.

*Significant findings at *P* < 0.05.

**Adj: adjusted; CI: confidence interval; LBW, low birth weight; NICU: neonatal intensive care unit; OR, odds ratio; RDS: respiratory distress syndrome; ROP: retinopathy of prematurity; RSV: respiratory syncytial virus.
